# G Protein-Coupled Receptor Kinase 2 Promotes *Flaviviridae* Entry and Replication

**DOI:** 10.1371/journal.pntd.0001820

**Published:** 2012-09-13

**Authors:** Caroline Le Sommer, Nicholas J. Barrows, Shelton S. Bradrick, James L. Pearson, Mariano A. Garcia-Blanco

**Affiliations:** 1 Department of Molecular Genetics and Microbiology, Duke University Medical Center, Durham, North Carolina, United States of America; 2 Center for RNA Biology, Duke University Medical Center, Durham, North Carolina, United States of America; 3 Duke RNAi Screening Facility, Duke University Medical Center, Durham, North Carolina, United States of America; 4 Institute for Genome Sciences and Policy, Duke University, Durham, North Carolina, United States of America; 5 Department of Medicine, Duke University Medical Center, Durham, North Carolina, United States of America; 6 Program in Emerging Infectious Diseases, Duke-NUS Graduate Medical School, Singapore, Republic of Singapore; Florida Gulf Coast University, United States of America

## Abstract

Flaviviruses cause a wide range of severe diseases ranging from encephalitis to hemorrhagic fever. Discovery of host factors that regulate the fate of flaviviruses in infected cells could provide insight into the molecular mechanisms of infection and therefore facilitate the development of anti-flaviviral drugs. We performed genome-scale siRNA screens to discover human host factors required for yellow fever virus (YFV) propagation. Using a 2×2 siRNA pool screening format and a duplicate of the screen, we identified a high confidence list of YFV host factors. To find commonalities between flaviviruses, these candidates were compared to host factors previously identified for West Nile virus (WNV) and dengue virus (DENV). This comparison highlighted a potential requirement for the G protein-coupled receptor kinase family, GRKs, for flaviviral infection. The YFV host candidate GRK2 (also known as ADRBK1) was validated both in siRNA-mediated knockdown HuH-7 cells and in GRK^−/−^ mouse embryonic fibroblasts. Additionally, we showed that GRK2 was required for efficient propagation of DENV and Hepatitis C virus (HCV) indicating that GRK2 requirement is conserved throughout the *Flaviviridae*. Finally, we found that GRK2 participates in multiple distinct steps of the flavivirus life cycle by promoting both entry and RNA synthesis. Together, our findings identified GRK2 as a novel regulator of flavivirus infection and suggest that inhibition of GRK2 function may constitute a new approach for treatment of flavivirus associated diseases.

## Author Summary

The *Flavivirus* genus includes several emergent and reemergent viruses, such as dengue and yellow fever viruses, which cause severe diseases in humans for which there is no approved treatment. Flaviviruses are transmitted to humans by arthropods and they rely on scores of vertebrate and invertebrate factors to replicate in these disparate hosts. Identifying the host factors involved in viral propagation is critical to understanding the molecular mechanisms of infection and the development of new therapeutics. To identify human host factors required for yellow fever virus propagation, we completed two genome-scale siRNA screens. Among the candidates discovered were the G protein-coupled receptor kinases GRK2 and GRK4. We focused on the protein GRK2, a kinase first identified for its role in cellular signal transduction. We found that GRK2 was a host factor needed for productive infection by yellow fever, dengue and hepatitis C viruses and was required for both viral entry and efficient replication of the viral genome. GRKs, which are considered druggable, may be used as targets to develop broadspectrum anti-flavivirals.

## Introduction

Although a century has passed since yellow fever virus (YFV), which gave flaviviruses their name, was shown to cause a human disease, flaviviral epidemics still represent a serious threat worldwide. Viruses such as dengue virus (DENV) and YFV have undergone a major geographic expansion in the past two decades [1], [2]. Currently, there are no approved treatments and few preventives for flaviviral diseases. Effective vaccines have been developed only against YFV, tick-borne encephalitis virus and Japanese encephalitis virus [3]–[5]. The YFV vaccine was generated in 1937 [6] and its use decreased occurrence of the disease significantly. Spread of the mosquito vector's range, increased urbanization, and, importantly, lack of vaccination in at-risk regions, however, has led to the reemergence of yellow fever [7]. Thus, there is a critical need for the development of anti-flaviviral treatments.

The molecular details of host-virus interactions taking place during flaviviral infection remain mostly unknown. Excitingly, the recent development of genome-scale siRNA screens to identify host factors required for viral propagation has shed light on the complexity of these interactions [8], [9]. We sought to identify host factors shared by different flaviviruses, as these may represent attractive targets for the development of broad-spectrum anti-flaviviral therapies. Thus, we conducted genome-scale siRNA screens to investigate human host factors required for YFV propagation and identified hundreds of candidate genes. Among these candidates are two members of the G protein-coupled receptor kinase family (GRK), GRK2 and GRK4. The GRK proteins have been primarily described for their role in G protein-coupled receptor (GPCR) phosphorylation leading to receptor desensitization [10], [11], but they also interact with, and presumably phosphorylate, dozens of non-receptor cellular factors [12], [13]. Here, we showed that GRK2 is required for efficient propagation of viruses from the *Flaviviridae* family and that a decrease in GRK2 level alters both virus entry and RNA genome amplification.

## Materials and Methods

### Cell lines

All cell lines were cultured at 37°C, 5% CO_2_ in Dulbecco's Modified Eagle Medium (DMEM, Invitrogen) supplemented with 10% Fetal Bovine Serum (FBS, Gemini). GRK2^−/−^, GRK2^−/−, bGRK2^ (which stably express bovine GRK2 in a GRK2^−/−^ background), GRK6 ^−/−^, and β-arrestin 1/2-KO MEFs were kindly provided by Dr. R.J. Lefkowitz (Duke University).

### Antibodies and western blotting

In immuno-fluorescence assays, DENV was detected with the anti-E mouse monoclonal antibody 4G2 (isolated from the DI-4G2-4-15 hybridoma, American Type Culture Collection) used at a 1∶2000 dilution. YFV-17D was detected with the mouse ascites fluid from animals harboring a hybridoma line produce after immunization with YFV (YF-mAF) (available through ATCC in 2005 under the reference NIAID V-525-701-562) used at a 1∶2000 dilution, or with anti-E antibody described above. HCV was detected with the anti-core primary antibody (C7-50, Abcam) used at a 1∶500 dilution. The Alexa488-conjugated anti-mouse secondary antibody (Invitrogen) was used at a 1∶2000 dilution. Nuclei were stained with Hoescht 33342 (Sigma).

For western blotting assays, proteins were extracted in 100 mM Tris-HCl pH 7.5, 4 mM EDTA, 1% Triton X100. GRK2 was detected using the GRK2 C-15 antibody (Santa Cruz Biotechnology) at a 1∶500 dilution. ß-actin was detected with the β-actin C4 antibody (Santa Cruz Biotechnology) at a 1∶4000 dilution. Western blots were analyzed using secondary antibodies and Odyssey infrared scanner from Li-Cor.

### Dengue replicon constructs and RNA templates

The dengue replicon constructs DRrep and DRrep-RdRPmut were a generous gift from Dr. E. Harris (UC, Berkeley) and are described in [14]. DRrep and DRrep-RdRPmut DNA constructs were digested by *XbaI* and purified using the QIAquick PCR purification kit (QIAGEN). RNA templates were produced by *in vitro* transcription using the MEGAscript T7 kit (Ambion) following the manufacturer's protocol for synthesis of capped RNAs. Few modifications were made: 2 µg of DNA template were used and the cap analog was m^7^G(5′)ppp(5′)A (New England BioLabs). The reactions were incubated for 6 hr at 37°C and subsequently treated with TURBO DNase for 15 min at 37°C.

The Fluc construct was created by insertion of the Fluc open reading frame, amplified by PCR from pGL3 (promega), into pTNT (Promega) using *XbaI* and *XhoI*. RNA templates were transcribed *in vitro* using the MEGAscript T7 kit following the manufacturer's protocol. All the transcribed RNAs were purified using the RNeasy kit (QIAGEN).

### Quantitative PCR analysis of viral RNA

Total RNA was isolated from infected HuH-7 cells using TRI Reagent (Sigma). cDNA was generated using High-Capacity cDNA Reverse Transcription kit (Applied Biosystems). Quantitative PCR (qPCR) was performed using Power SYBR Green PCR master mix (Applied Biosystems) and the StepOnePlus real-time PCR system (Applied Biosystems). The primers used to detect YFV-17D were 5′-GGGCGAAGGAGTATCCCAGT-3′ and 5′-ACGCTAACCAGCATCATCAGGAGT-3′. Each sample was normalized based on the amount of beta-actin detected using the primers 5′-GCTCGTCGTCGACAACGGCTC-3′ and 5′-CCTCGTCGCCCACATAGGAATC-3′. The relative amount of YFV-17D cDNA present in the different samples was calculated as described in [15].

### Viral stocks and virus titration

Preparation of DEN2-NGC and YFV-17D stocks has been previously described [8]. Infectious stocks of JFH1 HCV were generated and titrated by foci forming assay using HuH-7.5 cells as described in [16].

Titers of stocks and experimental samples were determined by foci forming assays. These assays were performed as described in Sessions et al. [8] with the following modifications: 2×10^5^ Vero cells/well were plated in 24-well plates. Following virus adsorption, 0.5 mL of 1∶1 1.2% Tragacanth Gum (Sigma)/2× EMEM (Lonza), supplemented with 5% FBS and 10 mM HEPES (Invitrogen) was added per well. The primary antibodies 4G2 and YF-mAF were used to detect DEN2-NGC and YFV-17D, respectively. Alexa488-conjugated anti-mouse antibody was used as secondary and foci were detected by immuno-fluorescence.

### siRNA screening and high content imaging

The protocol used to screen the Human genome siRNA library (QIAGEN) has been described in detail in Barrows *et al*. [17]. Briefly, HuH-7 cells were reverse transfected with a total siRNA concentration of 15.4 nM, each siRNA being present at 7.7 nM. Approximately 51 hr after transfection, the siRNA-treated cells were infected with YFV-17D at a M.O.I. of 0.1. Forty two hours post-infection, the HuH-7 cells were fixed with 4% PFA and stained with the 4G2 primary antibody followed by the Alexa488-conjugated secondary antibody. Each assay well was imaged using the Cellomics Array Scan VTI system (Cellomics). To determine the percentage of infection, the acquired images were analyzed using the vHCS Scan version 5.1.2.

When siRNAs are used individually, the same protocol as described above was used except that HuH-7 cells were reverse transfected with 7.7 nM of siRNA.

### Sum rank algorithm

22,909 genes were interrogated by the Qiagen Human Genome siRNA Library. 21,529 genes passed the criteria that the cell density in all 4 sets (GS1AB, GS1CD, GS2AB, GS2CD) exceeded our minimum threshold. Within each set the percent infection values were ordered low to high, and a rank was assigned. The ranks were summed for each gene generating the sum rank 4SR. The statistical limit to identify a putative YFV host factor from the genomic screen was set, a priori, at a p-value≤0.00135. 395 hits were identified as required for the propagation of YFV and are presented in Table S3. The 4SR values were normalized to the mean and standard deviation for a simulated population distribution and the Z-statistic and associated p-value are reported in Table S1. For a detailed description of the 4SR statistical interpretation see Figure S1.

### Virus entry assay

The entry assay was performed following protocols in [18], [19]. HuH-7 cells treated with siRNA control or siRNA targeting GRK2 for 48 hr were incubated with YFV-17D (M.O.I. = 5) at 37°C. After 2 hr cells were placed at 0°C and washed three times with chilled PBS. Cells were then incubated with chilled 1 M NaCl, 50 mM Na_2_CO_3_, pH9.5 for 3 min. This solution removes the surface bound virus without affecting the internalized virus. Cells were washed again three times with chilled PBS and finally treated with TRI Reagent (Sigma) to collect total RNA.

### RNA replicon electroporation and luciferase assays

HuH-7 cells treated with siRNA control or siRNA targeting GRK2 for 48 hr were trypsinized and wash once with DPBS (Invitrogen), and resuspend in 0.5 mL of OPTI-MEM I (Invitrogen) to a concentration of 5×10^6^ cells per mL. 15 µg of replicon RNA, 3 µg of Fluc RNA control and 80 pmol of siRNA were added to the cells. The mixture was transferred to a 0.2 cm cuvette (Bio-Rad) for electroporation and kept on ice. Electroporation was performed at room temperature using the Gene pulser II apparatus (Bio-Rad) set at 270 V and 200 µF. Immediately after the pulse, 1 mL of media was added to the cells. Later the electroporated cells were diluted in 11.5 mL of DMEM, 10% FBS media and 0.5 mL/well was plated in 24-well plate. At indicated times, cells were washed once with DPBS and lysed into 100 µL of passive lysis buffer (Promega). The luciferase activities were measured using the Dual-Luciferase Reporter assay kit (Promega) following the manufacturer's protocol. Rluc activity from the replicons was normalized to the mean of Fluc values detected during the first 26 hr.

### Statistical analyses

Statistical analyses were performed using GraphPad Prism 5.0 software as described in the figure legends.

## Results

### Genome-scale siRNA screens identified GRK family members as potential YFV dependency host factors

**Figure 1:**
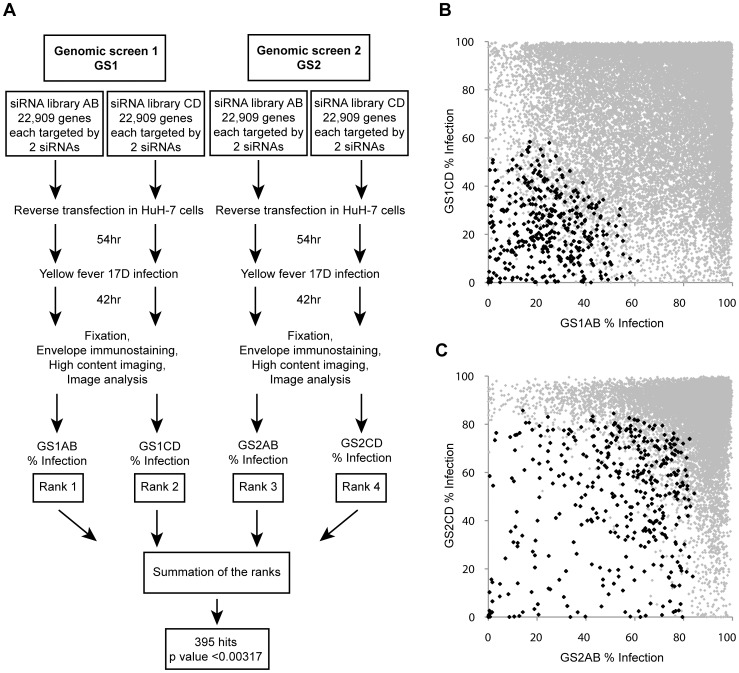
Genome-scale siRNA screens identify YFV human host factors. (A) Schematic of the protocol used in the siRNA screens and of the method used to identify hits. (B) Percentage of infection for the population in GS1AB (Genomic Screen 1, AB siRNA set) plotted against the percentage of infection for the corresponding wells within GS1CD (GS1, CD siRNA set). The black diamonds represent the identified hits and the grey diamonds represent the rest of the wells. (C) Similar to B, except that percentage of infection for the population in GS2AB (Genomic Screen 2, AB siRNA set) is plotted against percentage of infection for the corresponding wells within GS2CD (GS2, CD siRNA set).

To identify candidate human host factors required for efficient propagation of YFV, we performed two identical genome-wide siRNA screens. Performing duplicate screens, five months apart, also represented a unique opportunity to analyze the reproducibility of siRNA-based whole-genome screens, as reported in Barrows *et al*. [17]. The screens were done in HuH-7 cells using the YFV vaccine strain 17D (YFV-17D) as summarized in Figure 1A. Infected cells were visualized by immuno-fluorescence staining of the viral envelope protein and quantified using high-content imaging and analysis. We screened using a 2×2 pooled siRNA library format which has the advantage of interrogating each gene with two independent siRNA sets each containing two siRNAs (siRNA A and siRNA B (set AB); siRNA C and siRNA D (set CD)) (Figure 1A and [17]). As indicated in Figure 1A, the percent infection from the four siRNA sets, GS1AB, GS1CD, GS2AB and GS2CD, were each ordered lowest to highest and attributed a rank from 1 to n (n = total number of genes analyzed). For each gene, the four ranks were summed and genes having a low Sum Rank (SR) were considered as YFV host factor candidates. The use of 4SR analysis minimized the number of false positive off-target candidates since a gene having a low 4SR was likely the result of a gene scoring with both independent siRNA sets in both screens.

We used the 4SR described above to derive a hit list composed of 395 members (Table S1). Figure 1B and 1C show how candidate host factors distributed in the overall GS1 and GS2 population, respectively. In order to assess the quality of the primary screening data and of the 4SR analysis, we tested the four unique siRNAs (A, B, C, and D) individually for a subset of candidates present in this 395 hit list. The effect of four individual siRNAs targeting 98 candidate genes was assayed in triplicate. The ranks of the 98 genes tested are shown in Figure S2. Two siRNAs were used as negative controls: siGFP, which targets the transcript coding for the Green Fluorescent Protein (GFP), and a non-silencing control siRNA, siNSC, described by Quiagen as an siRNA with no homology to any known human gene. An siRNA was considered validated if it decreased the percentage of infection by at least 2 fold compared to infection observed with siGFP. The siGFP was used as a reference because it was the more conservative of the two negative controls: 76.6% infection in siGFP treated cells versus 84.5% infection in siNSC treated cells. In this validation, 88 out of 98 genes scored (89.8%) with two siRNAs or more showing a 2-fold decrease of the level of infection and an associated p-value≤5.8×10^−6^ (Table S2). This level of validation shows that when a 2×2 pooled siRNA library format is used, a high confidence list of gene candidates can be directly derived from the primary screen data. It should be noted that the validation rate for a particular candidate may vary depending on its SR (Figure S2).

We compared the 395 candidate host factors required for YFV propagation to those identified for WNV and DENV [8], [9]. We also considered a sub-genomic screen performed by Drs. P.W. Mason and F. Santa Maria Guerra, which interrogates a subset of 5500 genes to look for WNV host factors [20]. The commonalities between the siRNA screens looking at flavivirus host factors are presented in Table S3. Previously described host factors such as subunits of the vacuolar ATPase complex (V-ATPase) [21]–[23] were identified by all three different screens. Interestingly, members of the GRK family, mostly known for their role in GPCR phosphorylation, were also represented among the shared factors. GRK6 was identified as a WNV host factor and GRK2 and 4 were identified as YFV host factors.

### GRK2 is required for YFV-17D propagation in both human and murine cell lines

Both GRK2 and GRK4 were identified as YFV host factor candidates. To explore further the role of the GRK family in YFV-17D propagation, we focused our experiments on GRK2, which is ubiquitously expressed in contrast to mainly expression in testis for GRK4 [24], [25].

**Figure 2:**
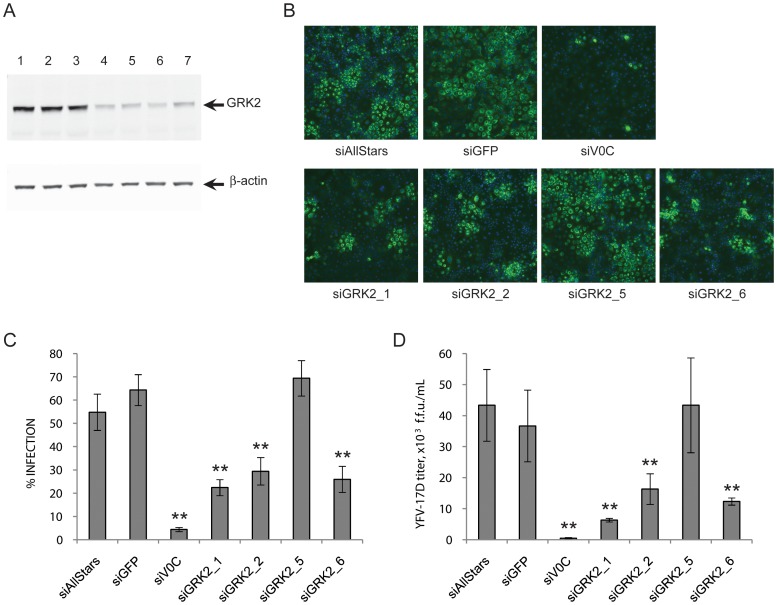
GRK2 is required for YFV-17D propagation in HuH-7 cells. (A) GRK2 levels assessed by western blotting in lysates of HuH-7 cells transfected with control siRNAs: siAllStars (lane 1), siGFP (lane 2), siV0C (lane 3), and with siRNAs against GRK2: siGRK2_1 (lane 4), siGRK2_2 (lane 5), siGRK2_5 (lane 6), and siGRK2_6 (lane 7). β-actin was used as a loading control. (B) Representative pictures of siRNA treated HuH-7 cells infected with YFV-17D at a magnification of 10× with the nuclei staining in blue and the viral staining in green. (C) Percentage of infected HuH-7 cells for the control and GRK2 knockdown conditions. The error bars represent the standard deviation of 10 wells. (D) YFV-17D infectious particles released from GRK2 silenced cells, indicated as foci forming unit per mL (f.f.u.. mL^−1^). Statistical significance was tested using an ANOVA test, siRNAs with an inhibitory effect with a p-value<0.01 compared to siAllStars control are indicated by **.

To confirm the screen results and to validate GRK2 as a YFV host factor, GRK2 was silenced in HuH-7 cells using the four siRNAs from the screened library individually. In this experiment, we used two negative control siRNAs: the siGFP and the siAllStars which is a newer version of non-silencing siRNA provided by Qiagen and that replaces the original siNSC described earlier. We also used a positive control siRNA, siV0C, which targets the transcript coding for ATP6V0C, a subunit of the V-ATPase complex previously shown to be required for flavivirus infection [8], [9]. The four siRNAs targeting GRK2 led to a 3 to 7 fold knockdown of the protein (Figure 2A, lanes 4 to 7) compared to the three siRNA controls (Figure 2A, lanes 1, 2 and 3). The siRNA treated cells were infected with YFV-17D at a M.O.I. of 0.1 and the level of infection was assayed by immuno-fluorescence after 42 hr. Representative images are presented in Figure 2B. The quantification of those images showed that, as expected, in cells transfected with the positive control siV0C, the level of infection was dramatically reduced (Figure 2C). In cells transfected with siGRK2_1, siGRK2_2 and siGRK2_6, the extent of YFV-17D infection was reduced by 2 to 3 fold compared to infection in cells treated with negative controls siRNAs. These observations validate GRK2 as a protein required by YFV-17D to replicate efficiently in HuH-7 cells. Unexpectedly, cells treated with siGRK2_5 in which the GRK2 protein level was decreased did not show a reduction of YFV-17D infection. This may be due to an off-target effect of this siRNA targeting not only GRK2 transcript but also a host factor which knockdown counteracts GRK2 knockdown effects.

To test whether the decrease of infection observed by immuno-fluorescence correlates with a diminution of infectious particle release, we measured the viral titer of culture media collected at the end point of the assay described above. Media from siRNA-treated HuH-7 cells infected with YFV-17D were used in focus forming assays. In the media from the GRK2 knocked down cells in which the YFV infection was altered, the viral titer was decreased by 2.4 to 6.3 fold compared to the control siRNA-treated cells (Figure 2D).

**Figure 3:**
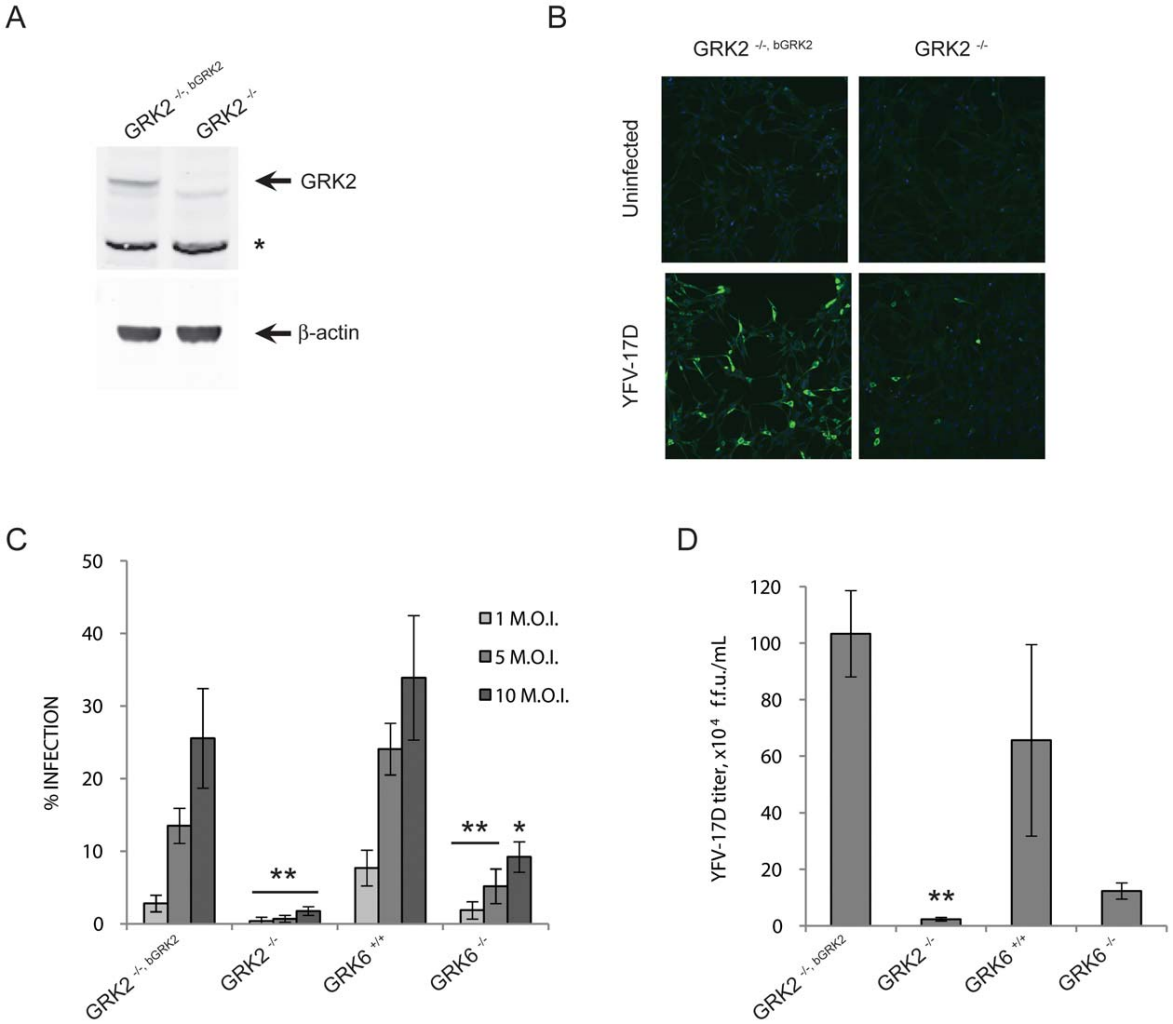
GRK2 is required for YFV-17D propagation in mouse embryonic fibroblasts. (A) GRK2 protein levels in GRK2^−/−, bGRK2^ and GRK2^−/−^ MEFs assessed by western blotting. β-actin was used as control. The asterisk indicates a non-specific band detected by the anti-GRK2 that is used as a second loading control. (B) Representative pictures of GRK2^−/−, bGRK2^ and GRK2^−/−^ MEFs infected or not with YFV-17D at a magnification of 10× with the nuclei staining in blue and the viral staining in green. (C) Percentage of infected cells for GRK2^−/−, bGRK2^, GRK2^−/−^, GRK6^+/+^, and GRK6^−/−^ MEFs. The error bars represent the standard deviation of at least 4 wells. (D) YFV-17D infectious particles released from GRK2 knockout MEFs, indicated as foci forming unit per mL (f.f.u.. mL^−1^). Statistical significance was tested using Welch's t-test, conditions with an inhibitory effect with a p-value<0.05 or <0.01 compared to control are indicated by * or ** respectively.

To further confirm a role of GRK2 in YFV infection, a siRNA-independent validation was also carried out using mouse embryonic fibroblasts (MEFs) in which the GRK2 gene was knocked out (GRK2^−/−^ MEFs). YFV-17D infection in GRK2^−/−^ MEFs was compared to infection in GRK2^−/−, bGRK2^ MEFs which stably express bovine GRK2 in a GRK2^−/−^ background (Figure 3A). These two cell lines were infected with YFV-17D at a M.O.I. of 1, 5 and 10. After 36 hr of infection the cells were immuno-stained for YFV-17D, and the percentage of infected cells was quantified. Representative images are presented in Figure 3B. In the GRK2^−/−^ MEFs, the infection was decreased by 7.8 to 19.8 fold at a M.O.I. of 1 and 5, respectively, compared to the infection in GRK2^−/−, bGRK2^ cells (Figure 3C). At the highest M.O.I. of 10, low level of infection could be detected in the GRK2^−/−^ cells, indicating that GRK2 was not absolutely essential for infection but was required for robust infection in MEFs. In media from GRK2^−/−^ MEFs, the viral titer was also reduced. A 45 fold decrease was observed between the GRK2^−/−, bGRK2^ and the GRK2^−/−^ MEFs (Figure 3D). Taken together, these results confirmed GRK2 as a YFV host factor both in human and murine cell lines.

### GRK2 is a host factor required by multiple members of the *Flaviviridae*

GRK6 was identified by Krishnan *et al*. as a WNV host factor [9] suggesting that members of the GRK family may be pan-flaviviral host factors. To test this hypothesis we asked whether GRK2 is also required for DENV propagation and if GRK6 is required for YFV-17D and DENV propagation.

**Figure 4:**
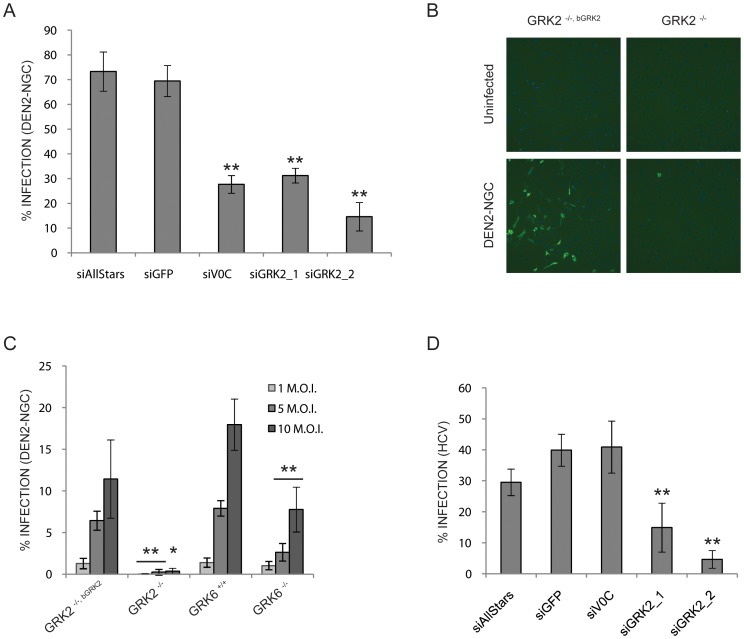
GRK2 is required for DEN2-NGC AND HCV propagation. (A) Percentage of infected cells for HuH-7 transfected with control siRNAs or with siRNAs against GRK2, and infected with DEN2-NGC. The error bars represent the standard deviation of 10 wells. Statistical significance was tested using an ANOVA test, siRNAs with an inhibitory effect with a p-value<0.01 compared to siAllStars control are indicated by **. (B) Representative pictures of DEN2-NGC infection in GRK2^−/−, bGRK2^ and GRK2^−/−^ MEFs at a magnification of 10× with the nuclei staining in blue and the viral staining in green. (C) Percentage of infected cells for GRK2^−/−, bGRK2^, GRK2^−/−^, GRK6^+/+^, and GRK6^−/−^ MEFs. The error bars represent the standard deviation of at least 4 wells. Statistical significance was tested using Welch's t-test, conditions with an inhibitory effect with a p-value<0.05 or <0.01 compared to control are indicated by * or ** respectively. (D) Percentage of infected cells for HuH-7.5 transfected with control siRNAs or with siRNAs against GRK2, and infected with HCV. The error bars represent the standard deviation of 10 wells. Statistical significance was tested using an ANOVA test, siRNAs with an inhibitory effect with a p-value<0.01 compared to siAllStars control are indicatedby **.

First, GRK2 was knocked down in HuH-7 cells using two validated siRNAs individually. As in the previous experiment, siAllStars, siGFP, and siV0C were used as siRNA controls. The siRNA treated cells were infected with DEN2-NGC 48 hr later. After 42 hr of infection, the cells were fixed and immuno-stained for the presence of E protein. Quantification of the infected cells showed a 2 to 5 fold decrease in cells treated with siGRK2 compared to cells treated with control siRNAs (Figure 4A). DENV2 infection was also measured in GRK2^−/−^ and GRK2^−/−, bGRK2^ MEFs. Both cell lines were infected with DEN2-NGC at a M.O.I. of 1, 5 or 10. Thirty-six hours later the cells were fixed, immuno-stained, and imaged (Figure 4B). In the GRK2^−/−^ MEFs the infection was decreased by 28 to 33 fold compared to the infection in the GRK2^−/−, bGRK2^ (Figure 4C).

Secondly, we tested another GRK, GRK6, which was required for WNV infection [9]. A GRK6 requirement was assessed using MEFs in which the GRK6 gene was knocked out, GRK6 ^−/−^ MEFs. GRK6 ^+/+^ MEFs derived from littermates were used as control. GRK6 MEFs were infected with YFV-17D or DEN2-NGC at a M.O.I. of 1, 5 or 10. Quantification of the infected cells after immuno-staining showed a 4 to 5 fold decrease of YFV-17D infection and a 2 to 3 fold decrease of DEN2-NGC infection in the GRK6 ^−/−^ MEFs (Figure 3C and Figure 4C). These results suggested that GRK6, like GRK2, was needed by both YFV and DENV to establish a high level of infection.

A recent siRNA “kinome” screen looking for factors required for hepatitis C virus (HCV) entry also identified GRK2 as a host factor [26]. HCV is a distinct member of the *Flaviviridae* that exclusively infects humans and establishes chronic infection of the liver. In order to verify this requirement in our experimental conditions, GRK2 was knocked down in HuH-7.5 cells [27] and the siRNA treated cells were subsequently infected with the replication-competent JFH1 strain of HCV [28]. After 72 hr of infection, the cells were fixed and immuno-stained for the presence of HCV core protein. Quantification of the infected cells showed a 2 to 8 fold decrease in cells treated with siGRK2 compared to cells treated with control siRNAs (Figure 4D). Of note, in contrast to YFV-17D and DEN2-NGC, we also observed that HCV infection did not require the ATP6V0C subunit to propagate.

In conclusion, our results indicated that the GRK family, specifically GRK2 and GRK6, was required for two different flaviviruses, YFV-17D and DEN2-NGC, to propagate efficiently in both human and murine cell lines. GRK2 was also required for HCV propagation in human cells. Taken together, our results suggested that GRK2 is required by multiple members of the *Flaviviridae*.

### GRK2 promotes viral entry by a mechanism that does not require β-arrestins

Decrease of infection observed upon reduction of GRK2 expression may be due to an alteration of virus entry, viral RNA translation, viral RNA synthesis, and/or infectious particle assembly and release. It has recently been proposed that GRK2 might be a host factor for HCV entry [26]. Therefore, to test whether GRK2 participates to the early steps of the flaviviral life cycle, we examined the ability of YFV-17D to enter HuH-7 cells treated with siRNA control or targeting GRK2.

**Figure 5:**
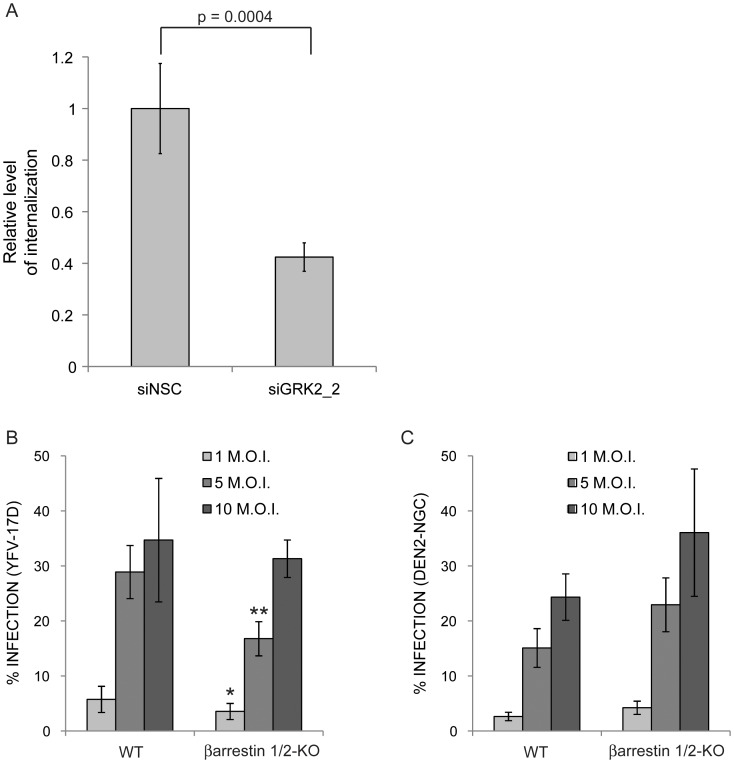
GRK2 plays a role in YFV-17D entry independently of β-arrestins. (A) HuH-7 cells treated with siNSC or siGRK2_2 were incubated with YFV-17D at a M.O.I. of 5 at 37°C for 2 hr. Samples were treated for 3 min with high-salt alkaline solution to remove the membrane-bound virus. Total RNA was then collected and YFV-17D genome was detected by quantitative RT-PCR. Each sample was normalized to the amount of beta-actin detected by qRT-PCR. The amount of YFV-17D genome detected is expressed in fold change over the control siNSC. Statistical significance was tested using Mann-Whitney non-parametric t-test and the p-value is indicated in the figure. (B and C) Percentage of YFV-17D (B) and DEN2-NGC (C) infected wild type (WT) and β-arrestin 1/2-KO MEFs. The error bars represent the standard deviation of at least 4 wells. Statistical significance was tested using Welch's t-test, conditions with an inhibitory effect with a p-value<0.05 or <0.01 compared to control are indicated by * or ** respectively.

To assess entry, HuH-7 cells treated with siNSC or siGRK2_2 were incubated with YFV-17D for 2 hr at 37°C which allows binding and internalization of the virus. To remove membrane-associated virus and ensure that only the internalized virus was measured, cells were treated with high-salt alkaline buffer. After additional washes, total RNA was collected and analyzed by qRT-PCR. This analysis indicated that the internalization of the virus was decreased by 2.5+/−0.5 fold in cells treated with siGRK2_2 compared to cells treated with siRNA control (Figure 5A). These results suggest that GRK2 plays a role in YFV-17D entry into HuH-7 cells. We cannot exclude, however, that the lower amount of YFV-17D RNA detected in cells treated with siGRK2 is the result of a lower stability of the viral genome when GRK2 is absent.

It has been previously shown that flaviviruses use clathrin-coated vesicles to enter cells [29], [30]. They share this property with GRK-phosphorylated GPCRs, which are internalized by a clathrin-dependent mechanism. After binding to ligands, GPCRs are phosphorylated by GRKs, and subsequently bind β-arrestins which interact directly with the clathrin complex and initiate receptor internalization [31]. In this process, β-arrestins are essential to clathrin-dependent internalization [11], [31], [32]. The arrestin family consists of four members but only β-arrestin 1 and β-arrestin 2 are ubiquitously expressed. Participation of β-arrestins in flavivirus infection was tested using MEFs in which both β-arrestin 1 and 2 genes were knocked out. Wild type (WT) MEFs derived from littermates were used as control. β-arrestin 1/2-KO and WT MEFs were infected with either YFV-17D or DEN2-NGC at a M.O.I. of 1, 5 and 10. After 36 hr MEFs were fixed and immuno-stained to assess infection. Quantification of infected cells showed no consistent differences between WT and β-arrestin 1/2-KO MEFs, with either YFV-17D or DEN2-NGC (Figure 5B and C). These results indicated that GRK2 mechanism of action in flaviviral entry is independent of the β-arrestins.

### GRK2 plays a role in multiple steps of the viral life cycle

**Figure 6:**
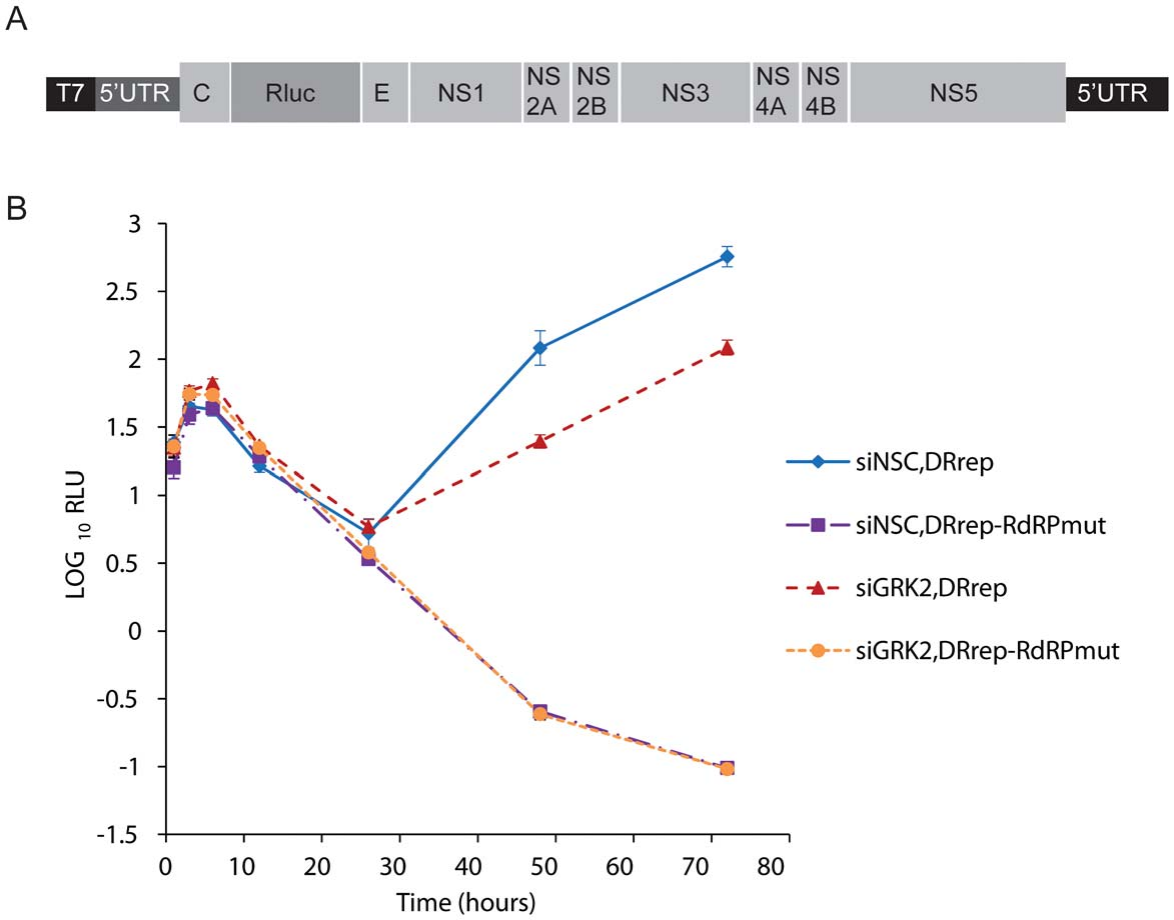
GRK2 plays a role in viral RNA synthesis. (A) Schematic of the DENV replicon construct which expresses Renilla luciferase (Rluc), adapted from Clyde *et al.* [14]. (B) HuH-7 cells treated with siNSC or siGRK2_2 were electroporated with DRrep and DRrep-RdRPmut RNA and assayed for Rluc activity at 1, 3, 6, 12, 26, 48, and 72 hours post-electroporation. Data represent the mean of relative light units (RLU) detected in triplicate samples. Error bars indicate the standard deviation and are sometimes hidden by the symbol.

To investigate whether GRK2 also plays a role in later stages of infection we used DENV replicons expressing renilla luciferase (Rluc) in place of the structural proteins (Figure 6A). Because replicons were electroporated and do not express the structural proteins, any differences observed upon GRK2 knockdown would be independent of changes in viral entry or assembly and egress of infectious particles. Wild type replicon (DRrep) or replicon which cannot synthesize RNA due to a mutation in the NS5 RdRP (DRrep-RdRPmut) were electroporated in HuH-7 cells treated with siNSC or siGRK2_2, and Rluc activity was measured overtime. DRrep-RdRPmut construct was used to assess translation of the input RNA. Early Rluc activity was similar in cells treated with control or GRK2 siRNA (Figure 6B) indicating that GRK2 knockdown did not affect translation of the incoming viral RNA. The same results were observed with the DRep replicon. GRK2 knockdown, however, resulted in a 5-fold decrease of Rluc activity at later time points, when newly synthesized viral RNA is produced. This result suggested that GRK2 plays a role at the level of viral RNA synthesis.

Altogether, these mechanistic data indicate that GRK2 acts as a key factor in propagation of flaviviruses by controlling multiple stages of the viral life cycle. Moreover, the assays utilized here suggest that GRK2 regulates viral entry and genome amplification independently.

## Discussion

We performed genome-scale siRNA screens to identify host factor candidates required for YFV-17D propagation in human cells. In recent years, there has been a concern that published hit lists from RNAi screens contain false positives due to sequence-dependent off-target effects. To address this concern, it was suggested that every published hit should be validated by at least two distinct siRNAs [33]. To meet this requirement, in most of the studies using RNAi screens, hits identified in a primary screen are submitted to a second round of screening using either alternative sequences or individual sequences if a siRNA pool was used [34]. In contrast, we chose a 2×2 pool screen format that interrogated each gene by two distinct siRNA pools at the first pass of screening. This screening format allows generation of gene candidate lists containing low number of false positives without having to run a second round of screening. It must be noted, however, that in case where only two of the four siRNAs are effective and happen to be in the same pool, the corresponding gene would not be considered as a candidate factor and this would be a false negative. Nonetheless, we posit that the early elimination of false positives outweighs the slight increase in false negatives, and we believe that the data produced using this screening strategy has generated a robust hit list of YFV host factors.

We previously found that hit list composition differs depending on the algorithm used to analyze data and on the defined threshold [17]. Therefore, it is valuable if entire datasets generated by siRNA genomic screens were made available to the scientific community for future re-analysis. To this end, we provide the entire datasets from two YFV screens as supplemental material (Table S1) so that other researchers can both assess and mine the data.

To our knowledge, this is the first time that the GRK family is validated as a *Flaviviridae* host factor and that GRK2 is described as a factor affecting the efficiency of both viral entry and viral RNA synthesis. Only the V-ATPase complex has been previously proposed as host factor able to control multiple stages of infection, release of the viral genome and egress of viral particles [22], [35]. This is new evidence that a single host factor can control independently distinct steps of the flaviviral life cycle. These two different functions in viral propagation might be explained by the ability of GRK2 to interact with and phosphorylate many different cellular proteins [36], [37].

GRKs are known as kinases that phosphorylate GPCRs such as the CC chemokine receptor 5 (CCR5) [38]. CCR5 is a well-described example of a human GPCR involved in viral infection and which mediates HIV-1 entry [39], [40]. Similarly, GRK2 could regulate a GPCR and therefore control YFV entry. GPCRs regulated by GRK2, such as the β2-adrenergic receptor [41], [42], or the P2Y purinergic receptors (P2YR) [43], were identified as YFV host factor candidates. Interestingly, the P2YR10 was also identified as a WNV host factor (Table S3, [20]). GRK2 function in entry could also be independent of receptor phosphorylation and could affect directly the endocytic machinery. In fact GRK2 interacts with various endocytic factors such as clathrin which was shown to be involved in DENV entry [29], [30], [44]. It would be interesting to test if the expression of a GRK2 mutant in which the interaction with clathrin is disrupted affects viral entry.

In this study, mechanisms that support viral RNA synthesis are also controlled by GRK2. Even though the translation of the incoming RNA is not affected by GRK2 knockdown, the translation of the newly synthesized RNA, which may require a different subset of factors, may be regulated by GRK2. It has been described that GRK2 can phosphorylate the ribosomal protein P2 (RPLP2) [45] which, together with the ribosomal proteins P0 (RPLP0), P1 (RPLP1) and L12 (RPL12), forms the stalk of the ribosome [46]. RPLP1 and RPLP2 are not required for general translation, but it has been suggested that they may regulate, depending on their phosphorylation state, the translation of a specific subset of transcripts [46]–[48]. We can hypothesize that the newly synthesized viral RNA may require phosphorylated RPLP2 to be efficiently translated. Intriguingly, we noted that RPLP1, RPLP2 and RPL12 were among YFV host factor candidates. It would be then interesting to test if RPLP1 and RPLP2 knockdowns affect the luciferase activity from the dengue replicon at late time points, as observed upon GRK2 knockdown.

In addition to providing mechanistic insights, future studies on the role of GRKs in flaviviral propagation could pave the way for much needed therapies to treat diseases caused by these viruses. The identification of a well-studied family of protein kinases as flaviviral host factor represents an attractive target for the development of anti-flaviviral drugs. Indeed members of the GRK family, which overexpression has been linked to heart failure, have been targeted by therapeutics for the treatment of cardiovascular diseases [49]. These drugs could represent starting points for the development of novel anti-flaviviral compounds.

## Supporting Information

Figure S1 **Population distribution for simulated, permuted and genomic 4SR distributions relative to normal distribution.** 22,909 genes were interrogated by the Qiagen Human Genome siRNA Library. 21,529 genes passed the criteria that the cell density in all 4 tests (GS1AB, GS1CD, GS2AB, GS2CD) exceeded our minimum threshold discussed in Barrows *et al*. [16]. Within each set the percent infection values were ordered low to high, and a rank was assigned. Ties received the same rank. The ranks were summed for each gene generating the summed rank 4SR. As shown along the X-axis, the lowest possible summation is a 4, while the greatest possible summation is 4*n (n = total number of genes analyzed) or 86,116. The cumulative probability value limit to identify a putative YFV host factor from the genomic screen was set, a priori, at a p-value≤0.00135. In order to determine the summation corresponding to this p-value, a computer simulation was performed. 1×10^8^ simulated summations were generated by summing four randomly selected whole numbers, each from a separate population of 21,529 units. The simulation identified the summation 9,150 as corresponding to a p-value equal at 0.00135. In order to establish that the simulation was accurate with respect to the actual data derived from the screening population, the ordered ranks were permuted with respect to each other and summed 1,008 times. The probability of obtaining a summation of 9,150 was equal to or less than 0.00137, demonstrating that the simulation was sufficiently accurate. 395 hits are identified in Table S1 as required for the propagation of YFV in human cells. Finally, the distribution of the simulated and permuted population distribution resembled, but was not identical to, a Normal distribution. Utilizing a Normal distribution provided the ability to assign readily interpretable statistics, so it was preferable to normalize the 4SR values using the mean and standard deviation of the simulated population, and report the Z-statistic and associated cumulative distribution. The mean and standard deviation calculated from the computer simulation was 43061.9 and 12430.2, respectively. The minimum and maximum summation of 4 and 86,116 correspond to a Z-score of −3.46 and +3.46, respectively. The summation 9,150 with a p-value estimated by the simulation to be less than or equal to 0.00135, has a Z-score of −2.73 with an associated p-value of 0.00318. Therefore, the Z-score approximation for the 395 hits will always underestimate the statistical significance demonstrated by the permutation test. As reported in Table S1, each gene is presented with the determined summation, Z-score as calculated by the aforementioned mean and SD, and the cumulative probability associated with the Z-score. Of note, the heavy tailed genomic population distribution was influenced by the covariance introduced by testing the same siRNA pool twice, as discussed in Barrows *et al.* [16]. Therefore, heavy tails do not necessarily represent meaningful biological enrichment.
(TIF)

Figure S2 **Validation by deconstruction of siRNA pools.** The axis represents the 395 ranked potential host factors. The vertical bars indicate when a factor was considered for deconstruction of siRNA pools. A gray bar indicates a validated hit, whereas a black bar indicates a hit that was not validated.
(TIF)

Table S1 **Complete data set for GS1 and GS2.**
(XLS)

Table S2 **Validation of 98 candidate hits: complete data set.**
(XLS)

Table S3 **Comparison of RNAi screens for DENV, WNV and YFV.**
(XLS)
