# Supplementary figures and images for: G Protein-Coupled Receptor Kinase 2 Promotes Flaviviridae Entry and Replication
Source: PLoS Negl Trop Dis. 2012 Sep 13;6(9):e1820. doi: 10.1371/journal.pntd.0001820 (PMC3441407; doi:10.1371/journal.pntd.0001820)

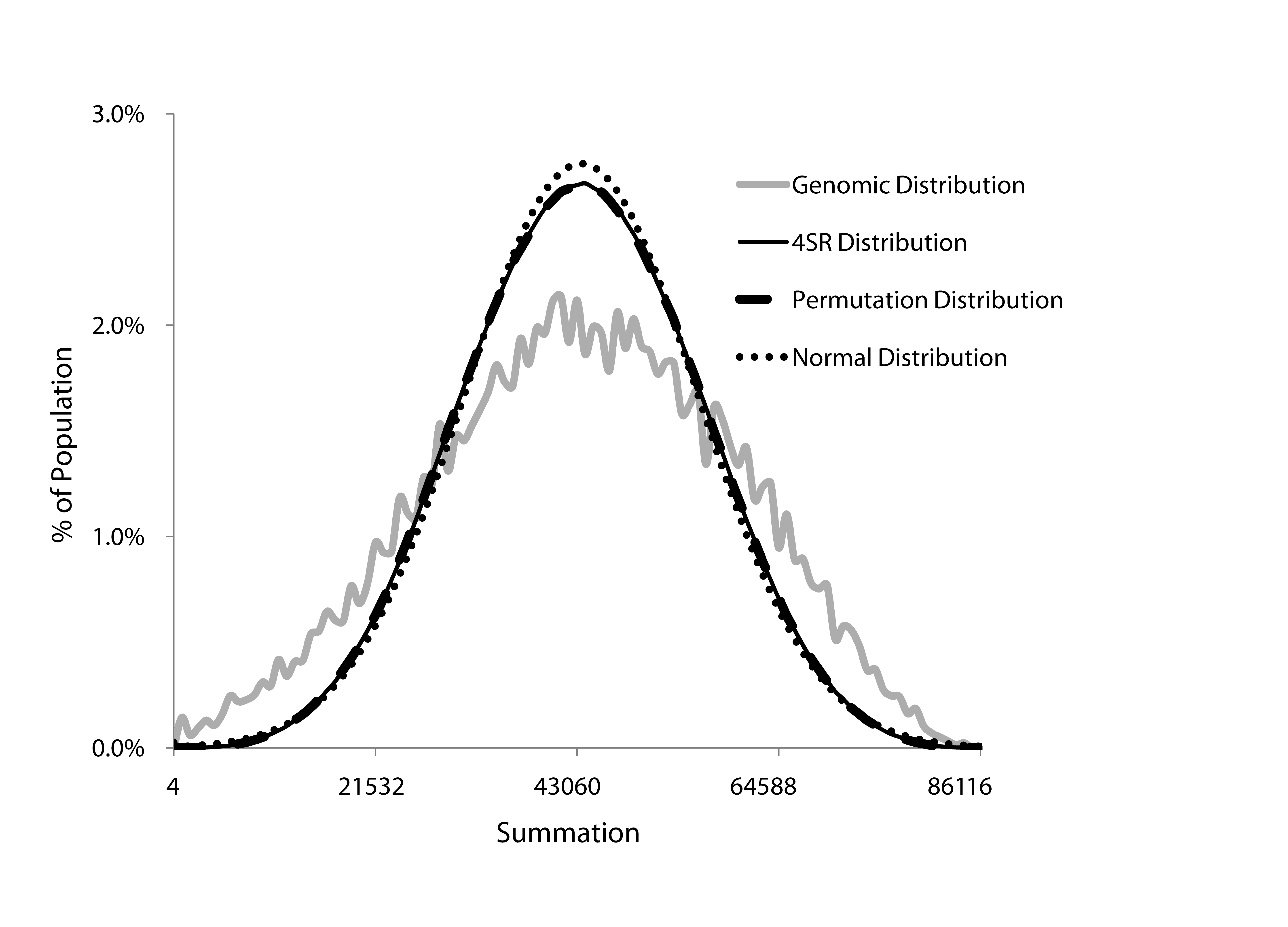

Supplement: Figure S1 — Population distribution for simulated, permuted and genomic 4SR distributions relative to normal distribution. 22,909 genes were interrogated by the Qiagen Human Genome siRNA Library. 21,529 genes passed the criteria that the cell density in all 4 tests (GS1AB, GS1CD, GS2AB, GS2CD) exceeded our minimum threshold discussed in Barrows et al. [16]. Within each set the percent infection values were ordered low to high, and a rank was assigned. Ties received the same rank. The ranks were summed for each gene generating the summed rank 4SR. As shown along the X-axis, the lowest possible summation is a 4, while the greatest possible summation is 4*n (n = total number of genes analyzed) or 86,116. The cumulative probability value limit to identify a putative YFV host factor from the genomic screen was set, a priori, at a p-value≤0.00135. In order to determine the summation corresponding to this p-value, a computer simulation was performed. 1×10^8^ simulated summations were generated by summing four randomly selected whole numbers, each from a separate population of 21,529 units. The simulation identified the summation 9,150 as corresponding to a p-value equal at 0.00135. In order to establish that the simulation was accurate with respect to the actual data derived from the screening population, the ordered ranks were permuted with respect to each other and summed 1,008 times. The probability of obtaining a summation of 9,150 was equal to or less than 0.00137, demonstrating that the simulation was sufficiently accurate. 395 hits are identified in Table S1 as required for the propagation of YFV in human cells. Finally, the distribution of the simulated and permuted population distribution resembled, but was not identical to, a Normal distribution. Utilizing a Normal distribution provided the ability to assign readily interpretable statistics, so it was preferable to normalize the 4SR values using the mean and standard deviation of the simulated population, and report the Z-stat [file pntd.0001820.s001.tif]

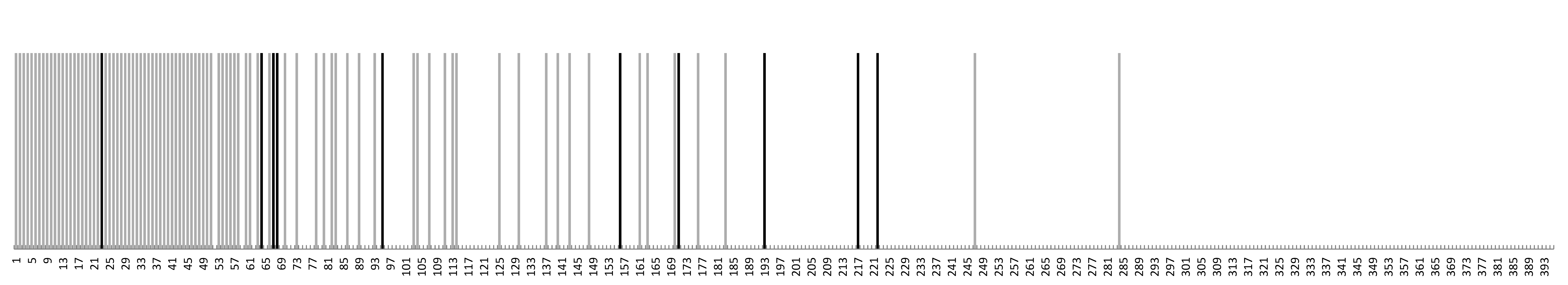

Supplement: Figure S2 — Validation by deconstruction of siRNA pools. The axis represents the 395 ranked potential host factors. The vertical bars indicate when a factor was considered for deconstruction of siRNA pools. A gray bar indicates a validated hit, whereas a black bar indicates a hit that was not validated. (TIF) [file pntd.0001820.s002.tif]
